# Patient Placement Criteria: Linking Typologies to Managed Care

**Published:** 1996

**Authors:** Leslie C. Morey

**Affiliations:** Leslie C. Morey, Ph.D., is a visiting associate professor of psychology at Harvard Medical School, Boston, Massachusetts, and an associate professor of psychology at Vanderbilt University, Nashville, Tennessee

**Keywords:** problematic AOD use, patient-treatment matching, patient care, managed care, patient assessment, disease severity, health insurance, treatment cost, disorder classification

## Abstract

The proliferation of managed health care systems as a means of controlling rising health care costs has stimulated efforts to subdivide the heterogeneous population of alcoholics into more homogeneous subgroups based on their needs for specific levels of treatment. The American Society of Addiction Medicine (ASAM) has developed a set of criteria aimed at helping clinicians select from four levels of care the one most appropriate for each patient. The ASAM criteria are designed around six criteria dimensions reflecting the severity of the patients alcohol-related problems. Although the ASAM criteria currently are the most widely used placement criteria for alcoholism treatment and reimbursement, they also have been criticized in several respects. Moreover, they still require outcome validation to ensure that application of the criteria improves treatment outcome.

For many years, alcoholism treatment providers predominantly assumed that people with drinking problems^1^ were a homogeneous group that could be treated optimally with only one treatment modality. This modality involved inpatient care with a fixed length of stay and a treatment approach based on the 12-step model of Alcoholics Anonymous. In recent years, however, both assumptions—that of patient homogeneity and treatment uniformity—have been abandoned. As the articles in this journal issue illustrate, researchers and clinicians now recognize that problem drinkers are a diverse group and differ substantially in the causes and manifestations of their alcohol-related problems. Furthermore, most researchers now believe that no single form of treatment is effective for all people presenting with alcohol-related problems (Hester and Miller 1989). Consequently, alcohol researchers now are conducting many studies de-signed to determine what types of interventions are most effective for what types of patients. This approach is founded on the “matching hypothesis,” which states that an optimal matching of patients and treatments will produce the greatest overall treatment effectiveness.

The need to acknowledge formally the heterogeneity of treatment needs among people with alcohol-related problems recently has received additional impetus from a direction unanticipated when the subtyping of alcoholics first became popular—namely, from the proliferation of managed care systems as a means of controlling health care costs (see sidebar, p. 38.). With the widespread use of managed care in treating alcohol and other drug (AOD) abuse in both the private and public sectors, the demand for specific types or levels of treatment (e.g., inpatient detoxification or residential rehabilitation) now depends on more than just the patient’s wishes or the physician’s perceptions of what the patient needs. Patients now must meet utilization review criteria set by the managed care providers in order to be eligible for treatment reimbursement. In addition to controlling costs, the development of such criteria will enable health care delivery systems to account for meaningful and valid differences among problem drinkers and to determine more accurately the mix of treatment services the patients need. Ultimately, the improved match between patient needs and the types of services available within the system will enhance the efficiency and effectiveness of the alcoholism treatment system. This matching process likely will focus on selecting specific treatment modalities rather than on the settings in which these modalities are provided.

**Managed Health Care**

The rapid development of managed care systems has resulted in sweeping changes in the U.S. health care system, including alcoholism treatment. Although the forerunners of such systems date back to the 1920’s (MacLeod 1993), the modern development of managed care accelerated during the 1970’s, stimulated by the private sector in response to years of unchecked inflation in health care costs and by widespread resistance to the concept of a national health insurance. The passage of both the Health Maintenance Organization Act in 1973, which required minimal benefits for alcohol and other drug abuse treatment, and subsequent amendments facilitated the expansion of the corporate practice of medicine. Large enrollment health care programs developed through new means of financing. These programs proliferated, with the expectation that competition between different programs would help contain health care costs with minimal government intervention.

One outgrowth of this movement was a major shift in the financing of health care. In many cases, individual fee-for-service payments were replaced by one prepayment covering services provided to each subscriber in the system for a specified period. This process is known as “capitation.” Under these new plans, health care providers had to bear part of the financial risk in providing services—for example, if providers incurred expenses exceeding the budgeted estimates, they had to absorb the deficit. This financing structure provided strong incentives to reduce hospital care and shift services to less expensive outpatient settings.

The term “managed” in “managed care” refers in part to the control that payers (i.e., insurance companies or health maintenance organizations) exert over health care decisions (e.g., which services an individual patient should use) in an effort to contain costs while ensuring adequate quality of care. This control often is accomplished through utilization reviews that include pre-admission certification of patients for in-patient care and concurrent reviews of patients in inpatient/residential care (and sometimes outpatient care) to determine whether the particular level of care is medically necessary. This process requires using placement criteria that clearly specify the requirements that patients must meet to be admitted to the various levels of care offered within the program. This requirement for placement criteria, however, significantly challenges the alcohol field, because to date no universally accepted criteria exist.

—Leslie C. Morey

ReferenceMacLeodGKAn overview of managed health careKongstvedtPRThe Managed Health Care HandbookGaithersburg, MDAspen Publishers1993

This article reviews the influences that led to the development of patient placement criteria as well as the process involved in designing such criteria. It summarizes the placement criteria developed by the American Society of Addiction Medicine (ASAM), which currently are the most widely used criteria, and presents both their advantages and disadvantages. Finally, the article describes the relationships of patient placement criteria with typological approaches based on patient characteristics.

## The Need for Patient-Treatment Matching

As researchers and clinicians began to explore the heterogeneity of individuals with drinking problems, their interest in differences between the various modalities and settings for treating alcohol-related problems also increased. One issue that stimulated much discussion concerned the merits of inpatient treatment for alcohol-related problems, and several studies concluded that inpatient treatment programs for alcoholism were no more effective than formal outpatient programs (Miller and Hester 1986; Annis 1986; Saxe and Goodman 1988). This conclusion forced the alcoholism treatment community to reexamine many of the basic assumptions that for years had dominated treatment programs.

The findings particularly raised questions about the cost-effectiveness of prevailing treatments. For example, Hayashida and colleagues (1989) demonstrated that inpatient detoxification of patients with mild to moderate features of alcohol withdrawal was no more effective than outpatient detoxification but costed approximately 10 times more. These results should be interpreted cautiously, however, because the study also found that treatment completion rates were higher in the inpatient program (95 percent) than in the outpatient program (72 percent).

Thus, although the overall effectiveness of inpatient care generally is accepted (Finney and Moos 1991; Walsh et al. 1991), concerns exist that this more costly approach has been applied to many patients who did not require this level of care. Moreover, aside from the actual dollar costs of treatment, other costs with potential clinical implications may be associated with inappropriate patient-treatment matching. For example, inpatient care disrupts the patient’s family life, employment, and social activities far more than does outpatient treatment.

The increasing influence of managed health care also has greatly stimulated patient-treatment matching considerations. In particular, managed care providers introduced utilization reviews that base treatment decisions on the patients’ abilities to meet certain criteria. The requirement for such reviews provided a significant challenge for the alcohol field, because until recently no criteria existed that had achieved widespread acceptance. The need to develop patient placement criteria prompted researchers, clinicians, and service providers to examine more closely existing alcoholism treatment modalities and their appropriateness for various subgroups of people with drinking problems.

## The Evolution of Patient Placement Criteria

As managed care became increasingly popular for both publicly and privately funded alcoholism treatment, insurance companies and various State agencies began to establish criteria to determine the appropriate placement or level of care for AOD abusers. These standards differed widely, however, and were often kept secret to prevent providers from slanting patient information in an effort to obtain more favorable outcomes during utilization review. In 1987 two organizations published guidelines that represented the initial steps toward generating more widely applicable criteria for establishing treatment-based typologies of problem drinkers. The Northern Ohio Chemical Dependency Treatment Directors Association developed a set of guidelines known as the “Cleveland criteria” (Hoffman et al. 1987) that used ratings in a variety of life areas to gauge the appropriateness of six levels of care. These levels ranged from mutual self-help groups to medically managed intensive inpatient units. The second organization, the National Association of Addiction Treatment Providers (NAATP), independently developed a similar set of criteria (Weedman 1987).

**Figure:**
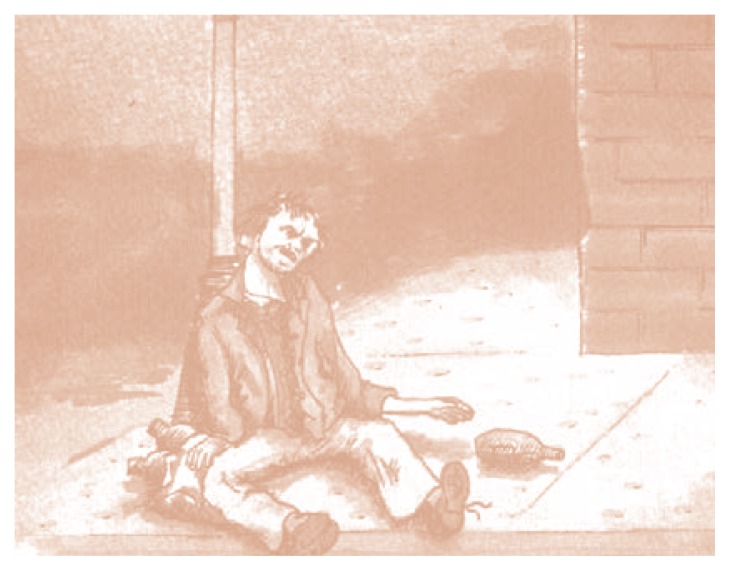
Type II/type B alcoholism illustrated in “Skid Row Bum.” Original artwork for “Temperance Tales and the Alcoholic,” 1979. Reproduced with permission from the *Journal of Studies on Alcohol*. © Alcohol Research Documentation, Inc., Rutgers University Center of Alcohol Studies.

Many experts who were involved in developing these two criteria sets also participated in task forces established under ASAM’s direction. These task forces developed a set of patient placement criteria that integrated and revised various features of both the Cleveland criteria and the NAATP criteria. These new guidelines, now called the ASAM criteria, were published by ASAM in a volume entitled *Patient Placement Criteria for the Treatment of Psychoactive Substance Use Disorders* (Hoffman et al. 1991). The criteria provide guidelines not only for alcoholism treatment but also for other forms of drug abuse and dependence.

### The ASAM Criteria

The ASAM criteria were developed from numerous and widely disseminated drafts and revisions and were evaluated in field tests at 15 different sites (Mee-Lee 1993). The primary goal of the criteria was to provide a common language for both providers and payers when determining the severity of a patient’s problems, the different levels or settings of the treatment modalities offered, and the criteria for patient placement within the continuum of AOD treatment. These criteria not only described patient characteristics that might warrant inpatient care but also provided guidelines for different types of outpatient treatment and outlined the process of moving across different levels of care.

The ASAM system is built around criteria dimensions that are used to place patients in one of four levels of care originally presented in an Institute of Medicine report (1990) describing transitions in the alcoholism treatment field. The four levels of care are as follows:

- *Level I: Outpatient treatment.* Such settings include organized nonresidential services or office practices in permanent facilities with designated addiction treatment personnel who provide professionally directed evaluation, treatment, and recovery services to addicted patients. The services are provided in regularly scheduled sessions of usually fewer than 9 hours per week.
- *Level II: Intensive outpatient and partial hospitalization treatment.* In these settings, an organized service with designated addiction personnel provides a planned treatment regimen consisting of regularly scheduled sessions of at least 9 hours per week within a structured program. This level of care affords patients the opportunity to interact with the real-world environment while still benefiting from a programmatically structured therapeutic milieu.
- *Level III: Medically monitored inpatient (residential) treatment.* These modalities, which are offered in permanent facilities with inpatient beds, include a planned regimen of round-the-clock professionally directed evaluation, care, and treatment for addicted patients provided by designated addiction personnel. The treatment is specific to AOD abuse and does not require the full resources of an acute-care general hospital.
- *Level IV: Medically managed in-patient treatment.* This level of care, which also is administered by designated addiction professionals, provides a round-the-clock planned regimen of medically directed evaluation, care, and treatment for addicted patients in an acute-care inpatient setting. Such a service requires permanent facilities that include, at a minimum, inpatient beds. A multidisciplinary staff and the full resources of a general hospital are available to provide treatment for patients with severe acute problems necessitating primary medical and nursing services. Treatment is specific to AOD-use disorders, although the available support services allow concurrent treatment of coexisting acute biomedical and emotional conditions.

Under the ASAM guidelines, patients are assigned to the four levels of care after being evaluated along six criteria dimensions reflecting the severity of the patients’ problems. Each dimension contains several criteria, and the number of specific criteria that must be met depends on the level of care. These six dimensions are described in the following paragraphs.

#### Dimension 1: Acute Intoxication and/or Withdrawal Potential

The ASAM criteria assume that a person who is acutely intoxicated cannot be monitored adequately as an outpatient and should receive more intensive care. When assessing withdrawal potential, one of the most important considerations is whether the patient is at risk of experiencing life-threatening withdrawal symptoms or requires medication or other support services to cope with or reduce the discomfort of withdrawal, which otherwise might cause him or her to terminate treatment.

#### Dimension 2: Biomedical Conditions or Complications

Higher levels of care are indicated when continued AOD use would put the patient in danger of health complications. For example, an alcohol-dependent woman who is pregnant might benefit from a higher level of care. Similarly, problem drinkers with cardiovascular, liver, or gastrointestinal diseases requiring medical monitoring or treatment should receive a higher level of care.

#### Dimension 3: Emotional and Behavioral Conditions and Complications

A wide range of emotional and behavioral conditions and complications exist in problem drinkers, either as manifestations of alcohol abuse or as independent, coexisting psychiatric disorders. These conditions (e.g., debilitating anxiety, guilt, or depression) deserve special attention during treatment and therefore may necessitate a higher level of clinical care. Moreover, problem drinkers exhibiting signs of an imminent risk of harming themselves (e.g., attempting to commit suicide) or others may require 24-hour monitoring, thus justifying a higher level of clinical care. The same holds true for problem drinkers whose mental status does not allow them to understand the nature of the disorder or the treatment process.

#### Dimension 4: Treatment Acceptance/Resistance

Patients in alcoholism treatment vary greatly in their willingness to comply with treatment regimens. Patients who seek treatment and cooperate by following clinical instructions typically require a lower level of care. However, alcohol dependence often compromises a person’s capacity to cooperate with treatment protocols. Patients often present for treatment with some level of understanding that alcohol is responsible for their alcohol problems but are still unwilling to participate in the clinical process. Other patients may deny that they have a drinking problem. Thus, some problem drinkers may be unlikely to enter the treatment system without first receiving some form of therapeutic preparation directed at addressing their denial and their resistance to treatment. Under these conditions, a high level of clinical care may be appropriate.

#### Dimension 5: Relapse Potential

Because alcohol-related problems involve recurrent patterns of behavior, relapse is a frequent and integral part of the natural history of the disorder. Two major sets of factors that derive from the patient’s personal (i.e., psychological and biological) background and social environment contribute to relapse potential. This dimension addresses the personal factors that influence the extent to which people can control their environments. (Environmental factors are addressed in dimension 6.) Accordingly, when these elements impede a patient’s control over his or her behavior in the current environment, a higher level of care (e.g., a halfway house rather than out-patient care) may be justified to minimize the relapse risk. For example, if a patient experiences marked and persistent cravings for alcohol and thus has higher relapse potential, treatment success may be less likely in an outpatient than in an inpatient setting.

#### Dimension 6: Recovery Environment

The patient’s environment can facilitate recovery or increase the risk of relapse. When the social setting is supportive (e.g., family members and friends agree with and encourage recovery) or the patient seeks out social surroundings that discourage alcohol-abusing behavior patterns, a lower level of clinical care may be justified. However, when a recovering person’s social setting is compromised—for example, by inadequate transportation to the treatment provider, a higher level of family stress, or friends and coworkers who regularly use alcohol—a higher level of care may be required.

Table 1 summarizes the correlations between the treatment settings and criteria dimensions specified by the ASAM guidelines. The actual criteria for placing an individual into a given level of care vary according to the care level, and placement ultimately depends on the combination of patient characteristics in the six assessment dimensions. For example, treatment in an outpatient setting (i.e., level I) requires that the patient meets level I criteria in all six assessment dimensions, whereas treatment in an inpatient setting (i.e., level III or IV) requires that the patient meets the corresponding severity criteria in at least two of the six dimensions. Furthermore, not all dimensions are relevant to all placement decisions. For example, treatment resistance, relapse potential, and recovery environment are not used to distinguish between patients requiring level III and level IV care.

## Advantages and Disadvantages of the ASAM Criteria

The ASAM criteria have become the most widely distributed and discussed criteria available, and several States have used them or some of their adaptations as guidelines for patient placement and medicaid reimbursement. Many aspects of the ASAM criteria make them reasonably well suited for such use. First, the criteria were developed by a multidisciplinary consensus group, including physicians, social workers, psychologists, and substance abuse counselors. Perhaps because of this comprehensive input, the dimensions composing the criteria relate to actual patient dispositions as well as to treatment dropout (Gastfriend et al. 1995). Second, the criteria have achieved much national visibility, far exceeding the impact of other criteria sets. Third, the ASAM guidelines separately consider factors influencing care for adults and for adolescents. This is important because the social factors associated with a need for higher treatment intensity may vary depending on age. Finally, the criteria specify guidelines for a broader continuum of care than traditionally found in the alcoholism treatment field. For example, the criteria include guide-lines for continued treatment at each level of care as well as for discharge eligibility. Thus, the ASAM criteria are among the few treatment-matching guidelines flexible enough to respond to changes in a patient’s status during the course of treatment.

Although the ASAM criteria currently are the most widely used patient placement criteria for treatment and reimbursement in the addiction field, they also have been criticized in several respects (Book et al. 1995; Gartner and Mee-Lee 1995). For example, relatively few studies to date have assessed the validity of the ASAM criteria, and although relevant projects currently are under way, no evidence yet exists that matching patients to treatments based on the ASAM criteria actually improves treatment outcome.

In the most relevant study, McKay and colleagues (1992) examined the validity of the Cleveland criteria, which were a precursor to the ASAM guidelines. The researchers studied alcoholic and cocaine-dependent patients who were treated in a day treatment program (i.e., ASAM level II). Assessment according to the Cleveland criteria revealed that 76 percent of these patients should have been assigned to inpatient rehabilitation (i.e., level III). The assumptions underlying the ASAM criteria would predict that patients who were “mismatched” to the level of care they received should have had poorer outcomes than those who were appropriately matched. However, the study found no differences in outcomes between matched and mismatched patients. Although the mismatched group drank slightly more frequently than the matched group before they entered treatment—consistent with the conclusion that severity of problem drinking is related to patient placement decisions resulting from the application of these criteria—the two groups did not differ significantly in their frequency of alcohol use after 4- or 7-month followup. Overall, all subjects appeared to respond well to treatment regardless of whether they were matched or mismatched according to patient placement criteria.

McKay and colleagues (1992) concluded that the Cleveland criteria may be overinclusive in identifying patients who require inpatient treatment. Similar criticism has been raised against the ASAM criteria, in part because the continuum of care represented in these guidelines does not include some important alternatives to traditional in-patient care (e.g., halfway houses or therapeutic communities). Other researchers have expressed concerns that the ASAM criteria overemphasize the medical elements of treatment and consistently place patients in higher levels of care than needed (Book et al. 1995).

The ASAM criteria also have been criticized for assuming that a linear relationship exists between the severity of the alcohol-related problems and the treatment level needed. For example, Book and colleagues (1995) indicate that in some patients, very severe problems (e.g., cognitive impairment or marked resistance to treatment) would impair their ability to benefit from expensive residential treatments. Consequently, these writers recommend that individuals who either are not yet ready for such treatments or not able to realize the benefits of residential treatment be treated in less expensive settings, such as halfway houses.

Other critics of the ASAM guidelines have noted significant conceptual and empirical gaps in the criteria, such as the lack of provisions for prevention and/or early intervention efforts (Gartner and Mee-Lee 1995). Moreover, the criteria do not cover several “sublevels” within each ASAM level. For example, intensive outpatient treatment and partial hospitalization (both regarded as ASAM level II) clearly differ in terms of treatment intensity. Similarly, halfway houses, therapeutic communities, and short-term intensive rehabilitation centers, which all can be considered residential treatment programs (i.e., ASAM level III), may vary widely in terms of treatment intensity.

Other gaps involve patient types rather than types of treatment settings. For example, Morey (1995) found that after strict application of the ASAM criteria, as many as 13 percent of individuals who met the criteria of the *Diagnostic and Statistical Manual of Mental Disorders, Third Edition, Revised* (DSM–III–R) for alcohol abuse or dependence did not meet the criteria for any of the four levels specified by ASAM. For instance, the ASAM guidelines commonly failed to include problem drinkers who were at risk for a problematic withdrawal but manifested no other biological, emotional, or psychosocial complications. These people did not meet the ASAM criteria for either outpatient treatment, because of their potentially complicated detoxification, or inpatient treatment (i.e., level III), which require problems in at least two of the six domains. Consequently, these patients were excluded from both outpatient and inpatient care.

## Relationships of Patient Placement Criteria to Other Typological Approaches

Unlike most of the earlier alcoholism typologies that were based on patient characteristics, the typologies inspired by the managed care movement focus on service systems. In other words, the traditional approach to subtyping people with drinking problems has involved identifying presumably fundamental differences among problem drinkers, followed by attempts to identify the most effective treatment for each sub-type. In contrast, the managed care-inspired typologies have focused on critical differences between the various forms of treatment, with a particular emphasis on the *costs* of different treatment approaches. In the interests of cost-containment and optimal allocation of resources, managed care has sought to develop patient placement criteria that restrict the most expensive forms of treatment—particularly inpatient services—to those patients who need them. The resulting subtyping strategy is treatment driven and typically arranged around treatment intensity. Consequently, these typologies often bear little resemblance to the typologies described elsewhere in this journal issue.

The common denominator under-lying patient placement typologies as well as many other patient-based typologies is general problem severity. Although the ASAM criteria themselves are multidimensional, the types of problems described across the six dimensions tend to be interrelated, and most studies demonstrate significant correlations between the level of care recommended according to the ASAM guidelines and global problem severity. For example, McKay and colleagues (1992) found a greater degree of psychological disturbance as measured by the Addiction Severity Index among patients identified as needing inpatient treatments than among those suited for outpatient treatment.

**Figure 1:**
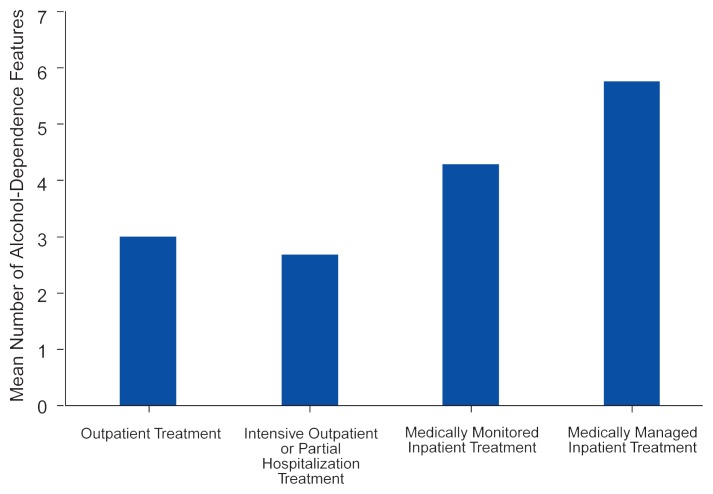
The correlation between the level of care of alcoholism treatment and the severity of the patients’ alcohol dependence. Based on telephone interviews with approximately 1,150 subjects from a community sample who met the criteria of the *Diagnostic and Statistical Manual of Mental Disorders, Third Edition, Revised* (DSM–III–R) for alcohol abuse and dependence, the researchers assessed the subjects’ severity of alcohol dependence and the level of care^1^ that they should receive during treatment. Dependence severity was indicated by the mean number of DSM–III–R criteria (of which nine were possible) that the subjects met. ^1^Level of care was determined according to the guidelines established by the American Society of Addiction Medicine. F = 38.14, *p* < 0.001. SOURCE: Adapted from Morey 1995.

In a study assessing the applicability of the ASAM criteria in a community sample, Morey (1995) used the guidelines to estimate level of care needs in a household survey of more than 18,000 people, roughly 6.5 percent of whom met the DSM–III–R diagnostic criteria for current alcohol abuse or dependence. The study related the number of alcohol dependence criteria met by the respondents (out of a possible nine) to the ASAM care levels that would have been assigned to these people. The results demonstrated that the number of dependence features reported by respondents who would have been assigned to inpatient treatments was considerably greater than for respondents who would have been assigned to outpatient treatment (figure 1). In fact, the respondents identified as needing intensive hospital care (i.e., ASAM level IV) on average fulfilled nearly seven DSM–III–R criteria, indicating severe dependence according to that diagnostic scheme.

The ASAM criteria’s focus on global problem severity when making placement decisions emphasizes quantitative differences among problem drinkers (i.e., people with more severe problems require greater treatment intensity). Many of the other alcoholism typologies, in contrast, have focused on qualitative differences (e.g., Cloninger’s type I versus type II classification or Babor’s type A versus type B classification; for more information on these other typologies, see the articles by Babor, pp. 6–14, and by Cloninger and colleagues, pp. 18–23). A review of the literature justifies careful consideration of the quantitative approach, which dates back to Jellinek’s four phases in the development of alcoholism that fell along a severity continuum (Jellinek 1962). Subsequent research has supported the contentions that features of alcohol dependence tend to form a unidimensional scale and that the cumulative severity of these dependence problems can predict relapse as well as posttreatment craving in abstinent individuals (Babor et al. 1988). This dimensional approach also forms the basis of the DSM–III–R definition of alcohol-dependence syndrome with its subdivisions of mild, moderate, and severe forms. Subtyping alcoholics along a continuum of alcohol-dependence severity therefore will capture many important differences among people with drinking problems. An exclusive focus on dependence severity, however, may overlook critical qualitative differences among problem drinkers that might assist in tailoring treatment approaches, particularly those offered within a given level of care.

**Figure 2:**
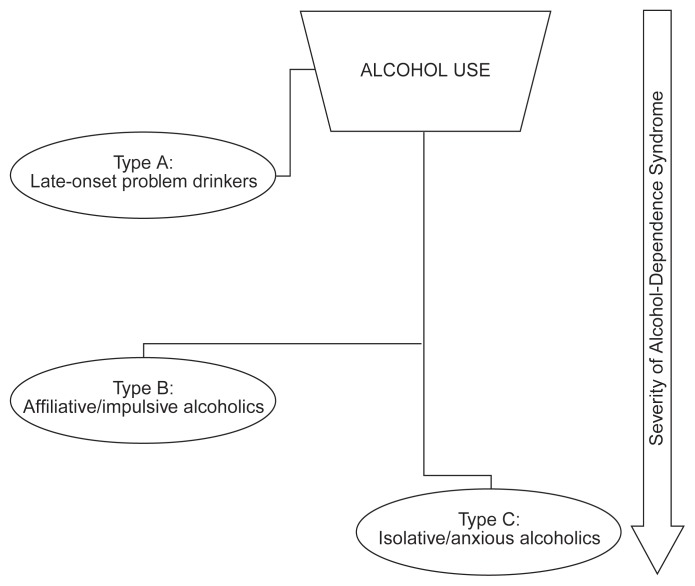
The hybrid model of alcoholic subtypes. This model distinguishes three categories of alcoholics: late-onset problem drinkers, affiliative/impulsive alcoholics, and isolative/anxious alcoholics. Late-onset problem drinkers differ in the severity of alcoholism (i.e., quantitatively) from drinkers in the other two categories. Affiliative/impulsive alcoholics and isolative/anxious alcoholics differ in certain personal characteristics (i.e., qualitatively) from each other. SOURCE: Adapted from Morey et al. 1984.

To avoid such a one-sided focus, Morey and colleagues (1984) developed the “hybrid” model, combining the quantitative and qualitative models of alcohol-related problems (figure 2). (For more information on this typology and how it was derived, see the article by Allen, pp. 24–29.) This typology distinguishes three types of people with drinking problems: late-onset problem drinkers, affiliative/impulsive alcoholics, and isolative/anxious alcoholics. The late-onset problem drinkers demonstrate significant signs of alcohol abuse but develop only mild manifestations of the alcohol-dependence syndrome. According to the ASAM criteria, nearly all these patients could be treated in outpatient programs. Late-onset problem drinkers differ quantitatively in their problem severity from the affiliative/impulsive and isolative/anxious alcoholics, who both are at an advanced level of alcohol dependence. Conversely, the latter two types differ qualitatively from each other with respect to interpersonal style, personality traits, concomitant symptoms of mental disorder, and specific features of their alcohol use. Consequently, although both affiliative/impulsive alcoholics and isolative/anxious alcoholics manifest severe dependence problems that likely necessitate relatively intensive treatment, they respond differently to the various elements of intensive treatment (e.g., group therapy as opposed to individual counseling) (Morey and Jones 1992). The hybrid model provides one example of how patient-based typologies can be integrated with the service-driven considerations that provide the foundation of patient placement typologies.

## Current Status of Patient Placement Criteria

The use of criteria, such as the ASAM standards, for guiding patients into different forms of treatment is a relatively new development in a rapidly evolving field. Although the ASAM criteria have been disseminated widely in the 5 years since their publication, they have not been uniformly accepted in either the public or private service delivery sector. Several States have revised the criteria to include treatment modalities that are not well covered by the ASAM guidelines (e.g., methadone maintenance for treating heroin addiction). Furthermore, the patient placement criteria used by managed care companies tend to be more restrictive than the ASAM guidelines regarding access to the more intensive levels of care (Book et al. 1995). These companies also typically separate the level II services outlined in the ASAM criteria, with partial hospitalization regarded as more intensive treatment with more extensive patient contact than intensive outpatient programs and therefore suitable for patients with greater problem severity.

A review of the current status of patient placement criteria by a consensus panel organized by the Center for Subtance Abuse Treatment (CSAT) suggested that the future acceptance of uniform patient placement criteria will hinge on several critical issues (Gartner and Mee-Lee 1995):

- The criteria should accurately reflect the different levels of care available.
- The criteria should have documented validity regarding recommended placement levels.
- The criteria should be easy to use in day-to-day clinical decisionmaking.
- The criteria should be measurable using reliable and objective tools.
- The criteria should encourage positive treatment outcomes by recommending treatments in the least restrictive environments.
- The criteria should optimally match patients to specific treatment modalities and levels of care.

The last point is particularly important because it addresses what is perceived as the inflexibility of current patient placement criteria. The CSAT panel recommended to “unbundle” the guidelines for the modality and intensity of treatment from the setting in which treatment is provided. Un-bundling means that the type and intensity of treatment are based more on the patient’s needs than on the setting in which they are provided; thus, psychiatric consultation could be offered in outpatient as well as inpatient settings, or intensive outpatient treatment could be offered in conjunction with a halfway-house setting. For example, large alcoholism treatment “campuses” might deliver different types of services all within one setting. Similarly, McGee (1995) has described a “human service matrix” in which the intensity of social support services needed (e.g., housing needs, child care, community support services, and occupational or legal assistance) is considered independently of the intensity of the clinical services provided (e.g., counseling or psychotherapy, nursing, or biomedical interventions). Separating some of the elements that appear inherently included in prevailing views of certain treatment settings would allow a much greater matching of specific interventions to different types of individual problems.

## Conclusions

Managed care undoubtedly has changed the alcoholism treatment field dramatically, and its influence will likely only increase in the near future. Although many concerns have been voiced about the effects of managed care on treatment, this new form of health care delivery presents extraordinary opportunities for research results to directly affect clinical practice. Managed care offers strong incentives to match patients with alcohol problems to the appropriate levels and types of treatment services, a goal that alcohol researchers have been striving to attain for the past two decades. Inherent in this movement will be a greater emphasis on evaluating outcome in different treatments. The array of data that will be collected in this area should help elucidate the fundamental distinctions among problem drinkers that are most relevant to treatment planning. Despite their outward—and, to some extent, concurrent—validity, however, placement criteria, such as the ASAM guideline, still require outcome validation to ensure that they are indeed related to differential treatment outcomes.

Concurrently, the evolution of patient placement criteria could be enhanced by carefully considering and integrating existing guidelines with the results of typological research. As the alcoholism treatment field moves toward unbundling treatment modalities from treatment settings, it also must understand the qualitative differences between individuals who may need treatments of similar intensity but with differing emphases. The literature on the subtyping of alcoholics includes numerous typologies that have assembled impressive evidence of validity. Ultimately, managed care may represent a means by which typologies demonstrating validity in the realm of treatment outcome can substantially influence alcoholism treatment practices in this country.

## Figures and Tables

**Table 1:** Summary of the ASAM^1^ Criteria Dimensions of Assessment

Criteria Dimension	Level I: Outpatient Treatment	Level II: Intensive Outpatient or Partial Hospitalization Treatment	Level III: Medically Monitored Inpatient (Residential) Treatment	Level IV: Medically Managed Inpatient Treatment
Acute Intoxication/Withdrawal Potential	Minimal to no risk of severe withdrawal; will enter detoxification if needed.	Minimal risk of severe withdrawal; will enter detoxification if needed and responds to social support when combined with treatment.	Risk of severe but manageable withdrawal, or has failed detoxification at lower levels of care.	Risk of severe withdrawal; detoxification requires frequent monitoring.
Biomedical Conditions	None or noninterfering with treatment.	May interfere with treatment but patient does not require inpatient care.	Continued use means imminent danger, or complications or other illness requires medical monitoring.	Complications (e.g., recurrent seizures or disulfiram reactions) that require medical management.
Emotional/Behavioral Conditions	Some anxiety, guilt, or depression related to abuse, but no risk of harm to self or others. Mental status permits treatment comprehension and participation.	Inability to maintain behavioral stability, abuse/neglect of family, or mild risk of harm to self or others.	Symptoms require structured environment, moderate risk of harm to self or others, or history of violence during intoxication.	Uncontrolled behavior, confusion/disorientation, extreme depression, thought disorder, or alcohol hallucinosis/psychosis.
Treatment Acceptance/Resistance	Willing to cooperate and attend treatment; admits problem.	Attributes problems externally; not severely resistant.	Does not accept severity of problems despite serious consequences.	Any difficulties noted in levels I, II, or III.
Relapse Potential	Able to achieve goals with support and therapeutic contact.	Deteriorating during level I treatment, or will drink without close monitoring and support.	Deteriorating and in crisis during outpatient care, or attempts to control drinking without success.	Any difficulties noted in levels I, II, or III.
Recovery Environment	Supportive social environment or motivated to obtain social support.	Current job environment disruptive, family/support system nonsupportive, or lack of social contacts.	Environment disruptive to treatment, logistic impediments to outpatient care, or occupation places public at risk if patient continues to drink.	Any difficulties noted in levels I, II, or III.

1 ASAM = American Society of Addiction Medicine.

## Footnotes

1 In this article, the terms “people with drinking problems” and “problem drinkers” refer to all individuals whose alcohol consumption has caused them medical, psychological, or social problems. These overarching terms therefore encompass the more medical diagnoses of alcohol abuse and alcohol dependence as defined by the American Psychiatric Association.
